# Effects of inbreeding on a gregarious parasitoid wasp with complementary sex determination

**DOI:** 10.1111/eva.12537

**Published:** 2017-10-13

**Authors:** Tania Zaviezo, Romina Retamal, Teddy Urvois, Xavier Fauvergue, Aurélie Blin, Thibaut Malausa

**Affiliations:** ^1^ Facultad Agronomía e Ingeniería Forestal Pontificia Universidad Católica de Chile Santiago Chile; ^2^ ISA INRA, CNRS, Université Côte d'Azur Sophia‐Antipolis France; ^3^ Université de Bourgogne Dijon France

**Keywords:** biological control, diploid males, hymenoptera, ichneumonidae, inbreeding depression

## Abstract

Inbreeding and inbreeding depression are processes in small populations of particular interest for a range of human activities such as animal breeding, species conservation, or pest management. In particular, biological control programs should benefit from a thorough understanding of the causes and consequences of inbreeding because natural enemies experience repetitive bottlenecks during importation, laboratory rearing, and introduction. Predicting the effect of inbreeding in hymenopteran parasitoid wasps, frequently used in biological control programs, is nonetheless a difficult endeavor. In haplodiploid parasitoids, the purge of deleterious alleles via haploid males should reduce genetic load, but if these species also have complementary sex determination (CSD), abnormal diploid males will be produced, which may jeopardize the success of biological control introductions. *Mastrus ridens* is such a parasitoid wasp with CSD, introduced to control the codling moth, *Cydia pomonella* (L.). We studied its life history traits in the laboratory under two conditions: inbred (full‐sib) and outbred (nonsib) crosses, across five generations, to examine the consequences of inbreeding in this species. We found that in inbred lines, nonreproducing females live less, the number of daughters produced was lower, and sex ratio (proportion of males) and proportion of diploid males were higher. Diploid males were able to produce fertile daughters, but fewer than haploid males. Lineage survival was similar for inbred and outbred lines across the five generations. The most significant decrease in fitness was thus a consequence of the production of diploid males, but this effect was not as extreme as in most other species with CSD, due to the fertility of diploid males. This study highlights the importance of determining the type of sex determination in parasitoid wasps used for biological control, and the importance of maintaining genetic diversity in species with CSD when importation or augmentation is the goal.

## 1 INTRODUCTION

The biology of small populations has important applications in areas such as species conservation (Hedrick & Kalinowski, 2000), animal breeding (Douglas, 1999), or pest biological control (Fauvergue, Vercken, Malausa, & Hufbauer, 2012). In the frame of classical biological control, natural enemies usually go through bottlenecks during their collection in the area of origin, during importation and culture in the laboratory, and most importantly, after release in the field. These bottlenecks can result in loss of genetic variability and increased inbreeding, which could be further exacerbated by the culturing methods, particularly when species are kept in laboratory rearing for long time (e.g., Francuski et al., 2014). High levels of inbreeding can result in inbreeding depression, the lower fitness of offspring of genetically related parents compared to that of unrelated parents (Charlesworth & Charlesworth, 1987; Charlesworth & Willis, 2009). Therefore, knowing the consequences of inbreeding in the natural enemies that are planned to be introduced for biological control is key to design the rearing and release methods that maximize population growth and demographic impact on target pests. For example, if inbreeding depression is severe, enriching laboratory colonies with wild individuals, multiple importations from the area of origin, or mixing populations from different origins could be a beneficial strategy (Francuski et al., 2014; Gujar, Chandrashekaran, & Kalia, 2006). However, adding individuals that differ genetically from those in rearing could also have no effect or even be detrimental (outbreeding depression; Benvenuto et al., 2012; Vorsino, Wieczorek, Wright, & Messing, 2012).

Among natural enemies, parasitoids of the order Hymenoptera are of particular interest for the study of inbreeding because most species have haplodiploid mode of sex determination, where diploid females are produced from fertilized eggs and haploid males develop from unfertilized eggs (Antolin, 1999; Heimpel & de Boer, 2008). In general, haplodiploid species are less affected by inbreeding than diploid species, because deleterious alleles are purged via the haploid males (Henter, 2003; Werren, 1993), although female‐limited deleterious traits, such as host searching or egg production, cannot be purged in this way (Antolin, 1999). Furthermore, many hymenopteran species within Ichneumonidae have complementary sex determination (CSD), which is probably the ancestral model of sex determination in the Hymenoptera (Asplen, Whitfield, de Boer, & Heimpel, 2009; Schmieder, Colinet, & Poirie, 2012). Under CSD, the ploidy level alone does not determine the sex of the individual, and the allelic composition at the sex locus (or loci) becomes crucial, with haploid (hemizygote) individuals being males, diploid heterozygote individuals being females, and diploid homozygote individuals developing into males (Heimpel & de Boer, 2008; Zhou, Gu, & Dorn, 2007).

Because in almost all hymenopteran species studied, diploid males are less viable (e.g., Petters & Mettus, 1980; Santomauro, Oldham, Boland, & Engels, 2004), sterile (e.g., El Agoze, Drezen, Renault, & Periquet, 1994; Fauvergue, Chuine, Vayssade, Auguste, & Desouhant, 2015) or produce nonviable daughters (e.g., De Boer, Ode, Vet, Whitfield, & Heimpel, 2007; Liebert, Sumana, & Starks, 2005), they are considered genetic dead ends, and hence represent a strong form of inbreeding depression (De Boer et al., 2007; Fauvergue et al., 2015). Compared to diploid species, haplodiploids with single‐locus CSD could be more susceptible to inbreeding depression and more extinction‐prone following perturbations, through a demographic–genetic feedback referred to as the “diploid male extinction vortex” (Zayed & Packer, 2005). Moreover, many species of Hymenoptera have life histories that could accelerate this extinction vortex by increasing the probability of producing diploid males and of females mating with them. For instance, gregarious immature development may increase the probability of sib‐mating, and viability of diploid males along with monandry may lower the fitness of sib‐mated females as well as females mating with diploid males (Godfray, 1994; Thiel & Weeda, 2014; Thiel, Weeda, de Boer, & Hoffmeister, 2013; Zayed & Packer, 2005). This puts strong pressure on Hymenoptera with CSD to develop ways to decrease sib‐mating, for example, through behavioral mechanisms (Metzger, Bernstein, Hoffmeister, & Desouhant, 2010; Ode, Antolin, & Strand, 1995; Van Wilgenburg, Driessen, & Beukeboom, 2006). However, in species where inbreeding is common, mechanisms that lower the cost of inbreeding may arise, such as diploid male fertility (Elias, Mazzi, & Dorn, 2009). Such mechanisms may nonetheless decrease the purging of recessive deleterious alleles underpinning phenotypic traits and make the effect of inbreeding more unpredictable than in diploid species or haplodiploid species without CSD.

*Mastrus ridens* Horstmann (2009) (Hymenoptera: Ichneumonidae) is a gregarious ectoparasitoid of *Cydia pomonella* (L.) (codling moth). It was collected in Kazakhstan and imported to the United States in the 1990s for biological control (Mills, 2005; Unruh, 1998), and more recently (2013 and 2015), it was collected in the same area and imported to Chile (Retamal et al., 2016; Zaviezo, Toleubayev, & Malausa, 2014). This species has been maintained for several years in laboratory colonies in different countries (Retamal et al., 2016; Sandanayaka, Chhagan, Page‐Weir, & Charles, 2011), where the sex ratio shifted from a female bias in earlier laboratory colonies in California (Bezemer & Mills, 2003) to a male bias in later New Zealand colonies derived from them (Sandanayaka, Chhagan, Page‐Weir, de Silva, & Charles, 2011). The existence of diploid males in laboratory colonies was suggested by Sandanayaka, Chhagan, Page‐Weir, de Silva et al., 2011 and evidenced by Retamal et al. (2016) using molecular markers and flow cytometry. Consistent with expectations, the frequency of diploid males is higher in laboratory colonies than in wild populations from its area of origin (Retamal et al., 2016). Therefore, *M. ridens* is a good candidate to study the consequences of inbreeding in a species with CSD and where inbreeding has a high probability of occurrence.

In this study, we performed controlled crossings between sibling and nonsibling individuals for five generations and monitored life history traits and male ploidy. Our first objective was to assess the effects of inbreeding in *M. ridens* on female life history traits and on the production of diploid males due to higher homozygosity at the *csd* gene. Our first hypothesis was that inbreeding would have little or no effect(s) on life history traits such as parasitism success, female fecundity, immature survival, and female longevity, mainly because in haplodiploid species most deleterious alleles are expressed and purged in hemizygous males. In case effects were detected, expression of deleterious alleles should increase over generations in inbred crosses because of the gradual increase of homozygosity. Our second hypothesis was that inbreeding would result in increased homozygosity at the CSD locus, generating diploid sons instead of daughters. As a result, the offspring will be constituted by fewer daughters and more sons (including diploids), leading to a more male‐biased sex ratio (larger proportion of sons). In this case, any negative effects of inbreeding would increase during the first few generations, as a consequence of the loss of CSD alleles, but would then remain stable once the lowest possible number of CSD alleles (two) is reached. A third related hypothesis, drawn by observations rather than theory, was that diploid males would have lower fitness than haploid males, and females that mate with them may produce fewer offspring, produce fewer females among their offspring, or produce partially fertile daughters. Our second objective was to evaluate the intensity of the impact of the CSD mechanism under conditions of inbreeding. Our fourth hypothesis was therefore that lines undergoing repeated sibling crossings would suffer higher extinction rates than lines undergoing outbred crossing at each generation, as a result of negative effects of inbreeding, including biased sex ratio caused by increased homozygosity at the CSD locus.

The results of this research are valuable for understanding the consequences of inbreeding and evolutionary adaptations in insects of the order Hymenoptera. From an applied perspective, our results should serve the optimization of rearing protocols and release strategies in biological control programs using parasitoids with CSD, starting with the particular case of *M. ridens* against the codling moth.

## 2 MATERIALS AND METHODS

### 2.1 Insect origin and rearing

A population of *M. ridens* was formed by mixing individuals descending from those collected in Kazakhstan in 2013 and 2014 with insects from the New Zealand mass‐rearing colony, imported to our laboratory in 2014, which in turn originated from insects collected in the same area in the 1990s by Mills and collaborators (Mills, 2005; Retamal et al., 2016). Mixing these populations in the past has resulted in populations with similar diversity to field populations in the area of origin (Retamal et al., 2016). Mated females were provided with cocooned fifth‐instar larvae of *C. pomonella* in pieces of corrugated cardboard, at a rate of one per day until death. Females were maintained in glass vials (2.5 cm diameter, 5.5 cm length) covered by a fine mesh, with a streak of honey as food. After 15 days from oviposition, *M. ridens* cocoons were separated and isolated in glass vials (1.5 cm diameter, 5.3 cm length) until adult emergence. This procedure was carried out to control mating and avoid sib‐mating, as females in the stock population were crossed randomly with nonbrothers. Environmental conditions were 25°C, 70% relative humidity, and 16:8‐hr light:dark photoperiod.

A colony of the host, *C. pomonella,* has been maintained in the laboratory since 2011, reared using an artificial diet (Stonefly Heliothis Diet; Ward's Natural Science, Rochester, NY), at 25°C, 70% relative humidity, and 16:8‐hr light:dark photoperiod. After 15 days in the diet, larvae were taken out and placed with pieces of corrugated cardboard (1.3 × 1.3 cm) as a substrate to spin a cocoon and pupate. The larvae used as hosts were kept for a maximum of 2 days at room temperature before being exposed to a female parasitoid. Larvae that continued the colony were left in the cardboard until pupation and then placed in cylinders (25 cm diameter, 80 cm length) covered in the inside with wax paper as oviposition substrate. Adult moths were fed with diluted honey spread in cotton.

### 2.2 Crossing treatments and insect management

From the stock population of *M. ridens*, each family was created using one female and one male. Using the progeny of these families (generation G0), two types of controlled crossing were carried out to produce the initial parental generation. Fifty females were mated with their brothers (sib‐mating) to form the inbred lines, and 52 were mated with males which were not a brother to form outbred lines. Crossing was repeated for five generations (G1 to G5). In inbred lines, inbred crosses were repeatedly performed at each generation to maintain or increase homozygosity over time. In outbred lines, outbred crosses were repeatedly performed to keep homozygosity as low as possible. At each generation, controlled crossings were performed as follows: One female and one male were placed in a glass vial (2.5 cm diameter, 5.5 cm length) for 12 to 24 hr, with honey as food. If at the time of female emergence males were not available, females were kept for a maximum of 2 days in the vial with honey at 25°C until a male emerged, which only happened in 5% of the cases. Males were mated only to one female.

After the mating period, the male was removed from the vial and preserved for genetic analysis (see below), and two cocooned fifth‐instar larvae of *C. pomonella* in pieces of corrugated cardboard were added. From then, host larvae were exposed to females at a rate of one per day until their death. Ten days after each host larva was exposed to a female parasitoid, they were checked for parasitism and, if present, *M. ridens* cocoons were counted. Five days later, cocoons were separated and isolated in glass vials until adult emergence, which occurred approximately 3 days later. To continue a line to subsequent generations, the first two daughters to emerge were collected and crossed according to the treatment (inbred or outbred) and provided with hosts as described above. If one of these females died before being provided with hosts, it was replaced with the next female to emerge (sister). This allowed us to have the progeny of two pairs per treatment per line and generation when enough females and males were available, but to continue the line to the following generation, the progeny of only one of them was used. Nevertheless, as generations passed, particularly in the inbred lines, few daughters emerged and either the progeny of only one daughter or none were available, and in the latter case, the line could not be continued. A total of 613 females were crossed and provided with hosts during the experiment.

Additionally, to measure female longevity in the absence of reproduction (not mated and no hosts provided), when possible, a third daughter was collected and kept in a vial with diluted honey as food. In total, the longevity of 247 females without reproduction was assessed, with numbers through generations and lines varying according to availability.

The whole experiment lasted around 5 months, with the generations of the different families more or less synchronous. Insects were kept at 25°C, 70% relative humidity, and 16:8‐hr light:dark photoperiod in a growth chamber. During the third generation, a fault caused the temperature in the chamber to increase briefly to 40°C, resulting in mortality of adult females. To minimize the impact of this event in the experiment, lines were continued using sisters that were in the pupal stage when this occurred.

### 2.3 Genetic diversity estimates

A sample of females from generations 1, 3, and 5, aiming to include the maximum representation of lines possible, was taken to estimate genetic diversity of inbred and outbred lines in time (Table S1). Additionally, we compared the genetic diversity of the lines used in this experiment with a field population sampled in Kazakhstan in 2015. In total, 258 females were genotyped using the 11 microsatellites reported by Retamal et al. (2016) and another six developed afterward (Table S2). Based on the genotyping data, we calculated the following: allelic richness (Na) using the rarefaction method (Kalinowski, 2005), observed and expected heterozygosity rates (Ho and He), and inbreeding coefficient (Fis). Observed and expected heterozygosities (Ho and He) were calculated with GeneClass2 (Piry et al., 2004) and allelic richness and inbreeding coefficients (Fis) with the R package “DiveRsity” (10,000 bootstrap iterations were used to calculate the 95% confidence intervals).

### 2.4 Female life history traits

To test whether inbreeding effects could be detected, the following traits were measured in inbred and outbred lines from G1 to G5: (a) parasitism rate, as the total number of parasitized larvae by a female in its lifetime divided by the total number of larvae exposed to it; (b) female fecundity, as (i) total adult offspring emerged (lifetime number of sons and daughters produced by a female), (ii) number of daughters, as total number of adult daughters produced by a female in its lifetime, and (iii) number of sons, as total number of adult sons produced by a female in its lifetime; (c) offspring secondary sex ratio, as lifetime number of sons divided by total offspring emerged; (d) immature survival (pupa to adult survival), as total adult offspring emerged divided by total number of parasitoid cocoons (egg and larva survival could not be measured directly without causing extensive undesired mortality); and (e) female longevity, as days from adult emergence to death, for (i) reproducing females and (ii) nonreproducing females.

### 2.5 Diploid male occurrence and male reproduction

To estimate diploid male occurrence, molecular analyses were carried out using a multiplex PCR kit with 11 microsatellite molecular markers developed for this species (Retamal et al., 2016). If a male was heterozygous at one or more markers, it was considered diploid. This estimation is conservative because diploid males homozygous for all microsatellite markers are erroneously categorized as haploid. The proportion of diploid males among those used for the controlled crossings in the G0 generation (*N* = 73) and for G1–G4 (*N* = 249) was estimated based on male origin data (crossing treatment and generation when the male was born). Generation 5 was not included, because only seven males from outbred lines were genotyped.

The progeny of females mated with genotyped males was available for 226 crosses. From this dataset, we determined the proportion of diploid (*N* = 71) and haploid males (*N* = 155) siring daughters (an indication of male fertility) under inbred and outbred crosses. Additionally, the number of daughters, number of sons, and total adult offspring in relation to male ploidy, crossing treatment, and generation were noted. Finally, to estimate whether males sired fertile daughters, we verified the proportion of diploid and haploid males siring granddaughters in inbred and outbred lines. This dataset included 12 diploid males and 108 haploid males that sired daughters for which their progeny were followed in the main experiment (i.e., there was a record of the progeny of the daughters). Note that because in the main experiment only up to two females were followed per line in each generation, the records of only up to two daughters per male were available.

### 2.6 Line survival

We defined a line as surviving across generations if the female used for trait measurements produced at least one daughter. We then recorded the generation to which each line survived (G1 to G5) and produced survival curves for the inbred and outbred lines. Note that lines considered as “extinguished” in this survival analysis were not necessarily lost for trait measurements (above), because whenever possible lines were continued with the backup female (sister), allowing trait measurement for the greatest possible number of generations for each line.

### 2.7 Data analysis

Female life history traits (Hypothesis 1). When data for two females per line per generation were available, only one was chosen at random for the analyses, which resulted in a dataset of the progeny of 266 crosses. Generalized linear models (GLM) with different error distributions and link functions were used to test the effect of crossing treatment (inbred or outbred), generation (1 to 5), and their interaction on life history traits. The best fitted models used a Poisson error distribution (accounting for overdispersion) with a log link function for count data (fecundity and longevity), and binomial error distribution (accounting for overdispersion) with a logit link function for proportional data (offspring survival and parasitism rate).

Traits associated with production of diploid males (Hypothesis 2). With the above dataset, we also used GLM to test for the effect of crossing treatment and generation on number of daughters (Poisson error distribution accounting for overdispersion with a log link function), offspring sex ratio (binomial error distribution accounting for overdispersion, logit link function), and proportion of diploid males (binomial error distribution, logit link function).

Diploid male fertility (Hypothesis 3). For this analysis, we used the data from all available genotyped males (*N* = 226) to increase representation of diploid males in inbred and outbred lines and generations. This included 157 crosses of the above dataset plus 69 from those not chosen for the female life history trait analysis. Similarly, we used GLM to test for the effect of male ploidy, crossing treatment, and generation on number of daughters, number of sons, and total offspring (Poisson error distribution accounting for overdispersion with a log link function), and the effect of male ploidy and crossing treatment on proportion of males siring daughters (binomial error distribution with a logit link function). Also, for the subset of males that sired daughters for which their progeny were followed in the main experiment (*N* = 120), the effect of male ploidy and crossing treatment on proportion of males siring granddaughters was analyzed (GLM binomial error distribution with a logit link function).

To analyze line survival (Hypothesis 4), Kaplan–Meier survival curves for inbred and outbred lines were generated and compared by the log‐rank method (Kleinbaum & Klein, 2012).

All GLM were tested with the anova.glm function and “F” test, except for the cases with binomial error distribution where the “Chisq” test was used, with R software version 3.2.1 (R Core Team, 2015). For multiple comparisons, when appropriate a contrast matrix was created with the contr.treatment function in R, and *p*‐values were adjusted using Holm method (Holm, 1979). All means in text and figures are followed by ± one standard error (SE), and details of all means and statistical analysis are presented in Tables S3–S5.

## 3 RESULTS

### 3.1 Genetic diversity estimates

Genetic diversity of populations at the beginning of the experiment was rather low: The observed heterozygosity rates were between 0.40 and 0.45 and allelic richness between 2.2 and 2.3 (Table 1). However, this extent of genetic diversity was similar to that of the field population sampled from the area of origin in Almaty, Kazakhstan (allelic richness: 2.16; observed heterozygosity rate: 0.39).

**Table 1:** Number of females genotyped and genetic diversity indices for inbred and outbred lines in three generations (G1 = generation 1; G3 = generation 3; G5 = generation 5), and a field sample from the area of origin

Treatment/generation	N	Na	Ho (SE)	He (SE)	Fis [CI]
Inbred/G1	42	2.23	0.434 (0.169)	0.417 (0.164)	−0.062 [−0.143; 0]
Inbred/G3	40	2.22	0.301 (0.151)	0.407 (0.183)	0.239 [0.120; 0.324]
Inbred/G5	29	2.18	0.249 (0.160)	0.407 (0.223)	0.349 [0.191; 0.499]
Outbred/G1	40	2.29	0.418 (0.142)	0.433 (0.160)	0.001 [−0.089; 0.070]
Outbred/G3	40	2.29	0.444 (0.192)	0.426 (0.173)	−0.053 [−0.143; 0.018]
Outbred/G5	40	2.23	0.412 (0.172)	0.420 (0.178)	−0.002 [−0.091; 0.067]
Kazakhstan sample	27	2.16	0.390 (0.239)	0.384 (0.205)	−0.047 [−0.155; 0.029]

N, number of individuals; Na, allelic richness estimate; Ho, observed heterozygosity; He, expected heterozygosity; SE, standard error; Fis, inbreeding coefficient; CI, 95% confidence interval.

During the experiment, only a little decrease in allelic richness was observed in both types of lines (from 2.23 to 2.18 in inbred lines and 2.29 to 2.23 in outbred lines), but inbreeding strongly increased in inbred lines over generations (Table 1). In inbred lines, observed heterozygosity (Ho) decreased from G1 to G3 (0.43 to 0.30) and from G3 to G5 (0.30 to 0.25) with inbreeding coefficient (Fis) increasing from −0.06 to 0.35. In outbred lines, observed heterozygosity rates kept over 0.41 and Fis were close to zero at the three generations (Table 1).

### 3.2 Effects of inbreeding on female life history traits

**Figure 1:**
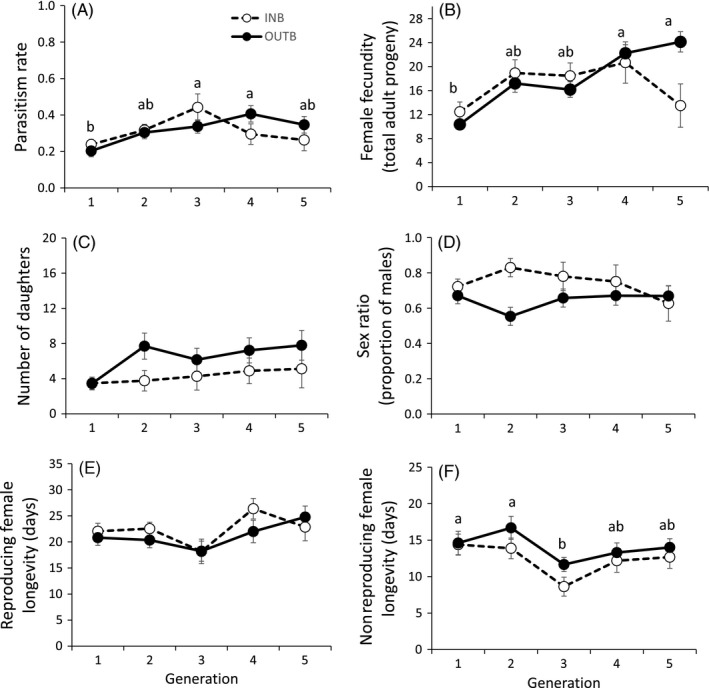
Female life history traits of *Mastrus ridens* under inbred (INB) and outbred (OUTB) crossing for five generations. (a) Parasitism rate: total number of parasitized larvae by a female in its lifetime divided by the total number of larvae exposed to it; (b) female fecundity: total adult progeny emerged (sum of sons and daughters produced by a female in its lifetime); (c) number of daughters: lifetime number of adult daughters produced by a female; (d) sex ratio (proportion of males): lifetime number of sons divided by total offspring; female longevity: days from adult emergence to death, for (e) reproducing females and (f) nonreproducing females. Error bars represent standard errors. Different letters correspond to significant differences between generation means (pooling inbred and outbred lines) according to multiple comparisons using a contrast matrix and Holm's *p*‐value adjustment

There were no effects of inbreeding on traits related to Hypothesis 1 (crossing treatment: *p* > .23 for all; crossing treatment x generation: *p* > .27 for all; Table S4), with the exception of longevity of nonreproducing females (crossing treatment: F_1, 168_ = 4.7; *p* = .032; Figure 1F). Nonreproducing females of outbred lines lived on average one more day than females of inbred lines (13.8 ± 0.6 versus 12.9 ± 0.7). Also, most traits showed a generation effect (Table S4). Parasitism was lowest in the first generation (generation: F_4, 260_ = 5.8; *p* < .001; Figure 1A). Fecundity increased through generations (generation: F_4, 222_ = 4.5; *p* = .002; Figure 1B), with total offspring emerged in the fifth generation being around twice that of the first generation (first generation 11.4 ± 1.2; fifth generation 21.5 ± 2.7). Adult longevity for reproducing females and nonreproducing females showed a decrease in the third generation, although this effect was only significant in nonreproducing females (generation: F_4, 169_ = 4.1; *p* = .008; Figure 1E‐F; Table S4). Pupa–adult offspring survival was 0.8 and independent of mating treatment, generation, or their interaction (*p* > .18 for all; Tables S3 and S4).

Inbreeding had a significant effect on number of daughters and lifetime offspring sex ratio (Hypothesis 2) (Table S4), with inbred lines producing fewer daughters and a more male‐biased sex ratio (higher proportion of sons) than outbred lines (Figure 1C–D; Table S3). Females of inbred lines produced on average 3.9 ± 0.5 daughters in their lifetime, while females of the outbred lines produced 6.1 ± 0.6 (crossing treatment: F_1, 221_ = 5.9; *p* = .016). The mean number of sons in a lifetime was larger than that of daughters, but similar for both types of lines (11.8 ± 0.9 sons for inbred and 10.8 ± 0.9 sons for outbred; crossing treatment: F_1, 221_ = 2.4; *p* = .12; Table S3). As with total offspring, number of sons increased with generation (generation: F_4, 222_ = 3.1; *p* = .017; Tables S3 and S4), with the exception of inbred lines in the fifth generation. Offspring sex ratio (sons over total offspring) was 0.74 ± 0.03 for inbred lines and 0.64 ± 0.02 for outbred lines (crossing treatment: F_1, 218_ = 9.4; *p* = .002), with no significant effect of generation (generation: F_4, 219_ = 0.1; *p* = .98; Figure 1D; Table S4).

### 3.3 Effect of inbreeding on diploid male occurrence and male reproduction

Inbreeding had a significant effect on proportion of diploid males (crossing treatment: χ^2^ = 4.1; df = 1; *p* = .043), with a higher proportion of diploid males in inbred lines agreeing with Hypothesis 2 (Table S3). Of the 322 males genotyped, 57 were heterozygote for at least one marker, and therefore unequivocally diploid. For males born in generations G1 to G4 that were genotyped (*N* = 249), 20.9% (23/110) of males in inbred lines were detected as diploid, while in outbred lines it was 11.5% (16/139).

**Figure 2:**
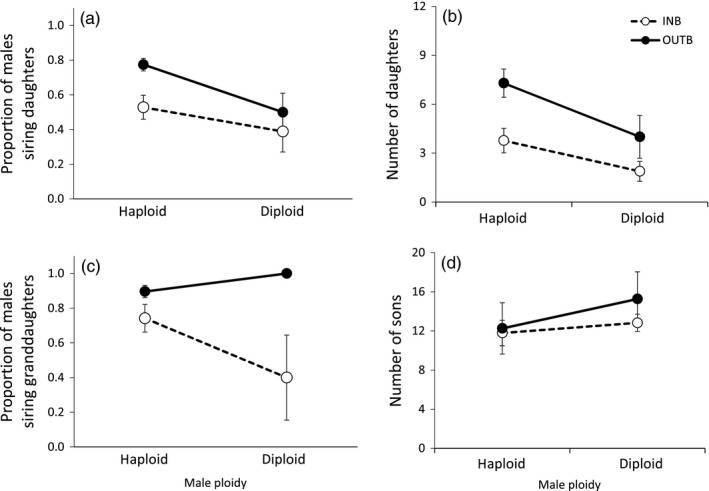
Male reproduction according to male ploidy for lines under inbred (INB) and outbred (OUTB) crossing for five generations. (a) Proportion of diploid males siring daughters: number of males siring daughters divided by total males; (b) number of daughters: lifetime number of adult daughters produced by a female when crossed to diploid or haploid males; (c) proportion of diploid males siring granddaughters: number of males siring granddaughters divided by total males siring daughters; and (d) number of sons: lifetime number of adult sons produced by a female when crossed to diploid or haploid males. Error bars represent standard errors

Regarding diploid male reproduction (Hypothesis 3), there was an effect of male ploidy (χ^2^ = 6.8; df = 1, 223; *p* = .009) and crossing treatment (χ^2^ = 12.5; df = 1, 224; *p* < .001) on the proportion of males siring daughters (Figure 2a, Table S5). In total, 45% (18/40) of diploid males sired at least one daughter compared to the 70% (130/186) of haploid males. When the type of crossing treatment was taken into account, we found that 53% (28/53) of haploid males with inbred crossing sired daughters and 77% (93/133) of haploid males with an outbred crossing sired daughters, whereas for diploid males these proportions were 39% (7/18) for inbred crossing and 50% (11/22) for outbred crossing (Figure 2a). Similarly, the total number of daughters sired by males was influenced by their ploidy (F_1, 219_ = 5.1; *p* = .024), crossing treatment (F_1, 220_ = 11.0; *p* < .001), and generation (F_4, 221_ = 2.9; *p* = .022), with no interaction among factors (Table S5). Diploid males sired on average 3.1 ± 0.8 daughters and haploid males 6.3 ± 0.5 daughters. In inbred lines, males sired 3.3 ± 0.6 daughters, while in outbred lines it was 6.8 ± 0.6 daughters (Figure 2b). Thus, the greater number of female offspring were sired by haploid males with outbred crossing and the least by diploid males in inbred crossing. Haploid males in inbred crossing and diploid males in outbred crossing sired similar number of daughters (3.8 ± 0.8 and 4.0 ± 1.3, respectively; Figure 2b). In the case of number of sons and total offspring, only generation had a significant effect (sons: F_4, 221_ = 2.7; *p* = .03; total offspring: F_4, 221_ = 4.2; *p* = .003; Tables S3 and S5), with fewer numbers in the first generation. There was also a trend for diploid males to produce more sons than haploid males (14.2 ± 1.9 versus 12.1 ± 0.7, respectively; F_1, 219_ = 3.1; *p* = .08) (Table S5, Figure 2d).

Of the 120 genotyped males for which we had records of their daughter's progeny, only 12 were diploid. A similar proportion of diploid (75%) and haploid males (85%) sired granddaughters (χ² = 6.8; df = 1, 118; *p* = .39; Table S5). Nevertheless, there was an effect of the crossing treatment on the proportion of males siring granddaughters (χ² = 7.4; df = 1, 117; *p* = .007), being 69% (25/36) for males in inbred lines and 91% (76/84) for males in outbred lines (Table S5, Figure 2c).

### 3.4 Effect of inbreeding on line survival

**Figure 3:**
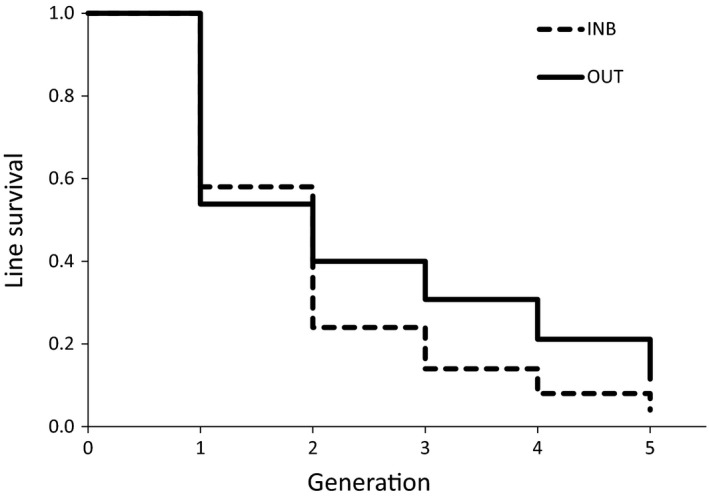
Kaplan–Meier survival curves for inbred (INB) and outbred (OUTB) lines across five generations

In both crossing types, lines were lost as generations passed, because a proportion of females in each generation produced no female progeny (daughters). In the inbred lines from the initial 50, only four lines (8%) reached the fifth generation when considering solely females used for the trait measurements, and in the outbred lines from the initial 52, only 11 lines (21%) were present by the fifth generation. On average, 53% of females of inbred lines produced daughters in each generation, compared to 66% of females of outbred lines (Figure 3). Nevertheless, in contrast to Hypothesis 4, inbred and outbred lines had similar survival trends through generations (survival log‐rank test χ² = 2.5; df = 1; *p* = .11).

## 4 DISCUSSION

In this study, we investigated the effects of inbreeding on *Mastrus ridens*, a gregarious parasitoid with single‐locus complementary sex determination, for five generations of repeated inbred and outbred crosses.

Our first objective was to study the effects of inbreeding on female life history traits. Although inbreeding depression is generally lower in haplodiploid species (Henter, 2003; Werren, 1993) because some deleterious alleles can be purged via hemizygous males, a range of effects on phenotypic traits, from insignificant to considerable, have been found in hymenopteran species (Antolin, 1999). In the sole study of inbreeding in a parasitic hymenopteran with known single‐locus CSD, egg load at female emergence appeared affected by inbreeding (Vayssade et al., 2014). Here for *M. ridens*, we found no evidence for inbreeding depression on life history traits such as parasitism rate, fecundity (total adult progeny), or immature survival, even after five generations of repeated inbreeding. Nevertheless, we did find a small but significant effect on longevity of nonreproducing females. Interestingly, in the review, made by Antolin (1999), of the three haplodiploid species where longevity was measured, there was an effect of inbreeding. In our study, some traits improved across generations (total number of offspring, number of sons, and parasitism), but without interaction with crossing treatment, suggesting a possible (nongenetic) adaptation to experimental conditions. An effect of generation was also found for female longevity, which was lower in the third generation, and can be a consequence of the unexpected temperature stress they suffered at that time, as temperatures of 35°C have been reported to decrease *M. ridens* longevity (Devotto, del Valle, Ceballos, & Gerding, 2010).

Our second focus was on the effects of inbreeding that could be associated with an increase in homozygosity at the CSD locus in *M. ridens* (Retamal et al., 2016). As expected, inbreeding yielded fewer daughters and consequently a sex ratio bias toward males. Also a higher proportion of diploid males was found in inbred lines compared to outbred lines (20.9% versus 11.5%, respectively). This difference could potentially be greater because heterozygosity at microsatellite loci was lower in inbred lines, and males homozygous for all markers were not classified as diploids (as a reference, 100% of females in outbred lines were heterozygous for the microsatellite markers compared to 96% in inbred lines). The proportion of diploid males estimated for inbred and outbred lines of *M. ridens* in this study is within the range we have found in our laboratory colonies, which have different rearing histories, with up to 25% diploid males for a population with long history of rearing in captivity and repeated bottlenecks (Retamal et al., 2016). On the other hand, the proportion of diploid males was only 4.2% in a field sample from the area of origin of *M. ridens* (Retamal et al., 2016), suggesting that our colonies may have a reduced number of CSD alleles compared to natural populations.

In haplodiploid hymenopterans, when diploid males are viable and able to mate, they are generally sterile and sire no daughters (e.g., Fauvergue et al., 2015; Garófalo & Estevam Kerr, 1975; Holloway, Heimpel, Strand, & Antolin, 1999), or when they do, they produce triploid and nonviable daughters (e.g., De Boer, Kuijper, Heimpel, & Beukeboom, 2012; De Boer et al., 2007; Liebert et al., 2005). We show here that in *M. ridens*, diploid males can reproduce and sire fertile daughters. However, diploid males were not as fit as normal haploid males: In our experimental conditions, 55% sired no daughters (against 30% for haploids), and those that succeeded produced half the number of daughters compared to haploid males. Surprisingly, the daughters of diploid males could produce some female offspring similarly to the daughters of haploid males. This suggests that females sired by diploid males are also diploid and have normal fertility, which is supported by the total absence of triploid individuals in genetic analyses (Retamal et al., 2016). This aspect of inbreeding deserves further, well‐designed studies, to complement the relatively few individuals we could track back a posteriori. Nevertheless, this represents the first reported case of fertility in diploid males for the family Ichneumonidae, and the third within the order Hymenoptera. The previous cases of diploid males with “full fertility” are the hunting wasp *Euodynerus foraminatus* (de Saussure) (Vespidae) (Cowan & Stahlhut, 2004) and the parasitoid *Cotesia glomerata* (L.) (Braconidae) (Elias et al., 2009). Both of these species have gregarious immature development (within a nest or host) and a life history that promotes inbreeding (Elias, Dorn, & Mazzi, 2010a; Stahlhut & Cowan, 2004). In these two species too, although diploid males sire fewer daughters compared to haploid males, they are not considered genetic dead ends (Cowan & Stahlhut, 2004; Elias et al., 2009). The mechanisms underpinning the lower number of female offspring in crosses involving a diploid male are unclear. For instance, it could be related to some degree of failure in mating or egg fertilization (Harpur, Sobhani, & Zayed, 2013). Anyhow, our results along with those of Cowan and Stahlhut (2004) and Elias et al. (2009) suggest that diploid male fertility could be more common than previously thought.

The effect of inbreeding on male reproduction echoed with that of male ploidy. In inbred lines, half of the males reproduced successfully and produced half the number of daughters compared to 3/4 successful males in outbred lines. Similarly, inbreeding significantly reduced the probability of siring granddaughters from 90% in outbred crosses to 70% in inbred crosses. Inbreeding reduces the success of producing daughters in *M. ridens* probably because diploid sons are produced instead of daughters (Harpur et al., 2013).

Given the effects of inbreeding identified in species with single‐locus CSD (more diploid males, fewer daughters, male‐biased sex ratio), we anticipated that inbred lines would go extinct faster than outbred lines, but this was not the case. A similar result was reported for *Cotesia glomerata*, a species with CSD and fertile diploid males, where similar extinction rates were observed in experimental populations initiated with different genetic variability and presumably different production of diploid males (Elias, Dorn, & Mazzi, 2010b). Similar extinction dynamics suggests either that the negative influence of inbreeding was small (36%–50% fewer daughters, 10% increase in sex ratio and proportion of diploid males) or that five generations are too few for the observed trends to be significant. In addition, the experimental design did not allow for more realistic population‐level effects on line survival, given that we only followed the progeny of isolated pairs and under very benign conditions (optimal temperature, hosts, and food in excess). Inbreeding depression is better revealed in stressful environments, as evidenced in insects such as *Tribolium castaneum* (Coleoptera: Tenebrionidae) (Hufbauer, Rutschmann, Serrate, Vermeil de Conchard, & Facon, 2013) and *Drosophila melanogaster* (Diptera: Drosophilidae) (Reed, Lowe, Briscoe, & Frankham, 2003; Woodworth, Montgomery, Briscoe, & Frankham, 2002).

The relatively small effects found for *M. ridens* in our inbred laboratory lines could be in part due to the existence of already some level inbreeding in our mass‐rearing, even after mixing different populations. Nevertheless, this level of inbreeding might not be unrealistic in comparison with what is found naturally in the field, and the genetic analyses showed that lines at the beginning of the experiment had similar heterozygosity and inbreeding coefficients than the 2015 field‐collected population, and also to the population collected in 2013, as well as mixed laboratory populations (Retamal et al., 2016). Our observations in the area of origin show that *M. ridens* is present in low numbers and with low parasitism rates, suggesting that this species may face chronically low populations, and due to its biology (gregarious immature development, viable diploid males, and monandry), inbreeding might be common in natural conditions. Moreover, the existence of highly fertile diploid males in a species with CSD would be compatible with a situation where inbreeding could not be avoided. These biological characteristics could also explain why this species has been successfully cultured for decades, despite recurrent bottlenecks. Nevertheless, our results also show that recurrent inbreeding in *M. rides* has an effect on heterozygosity and that if successful laboratory colonies need to be developed, efforts to promote outbreeding should be made.

To sum up, this study suggests that the effects of inbreeding on *M. ridens* are mainly due to the production of diploid males, because the effects of inbreeding were detected on traits such as proportion of diploid males, number of daughters, and sex ratio, and not on other relevant life history traits. Moreover, the negative impact of inbreeding in *M. ridens* is not as extreme as that predicted by earlier models for haplodiploid species with sterile diploid males (Fauvergue et al., 2015; Zayed & Packer, 2005).

The results of this research have important implications for both classical and augmentative biological control programs using parasitoid wasps. In classical biological control, natural enemies are sampled in the pest's native range and imported and cultured before being introduced at low numbers in the target environment (Van Driesche, Hoddle, & Center, 2008). Augmentative biological control relies on the mass‐rearing of natural enemies in large numbers and involves a commercial setting and well‐planned mass releases in agricultural landscapes (Van Driesche et al., 2008). Although these successive steps of bottlenecks and adaptive challenges should have dramatic consequences on the genetic structure of candidate biological control agents, dedicated studies are rare. Yet, better knowledge on the genetics of biological control agents could serve in guiding collections, optimizing rearing, and insuring population establishment (Hopper, Roush, & Powell, 1993; Hufbauer & Roderick, 2005; Roderick & Navajas, 2003). For parasitoids with CSD, efforts should focus on the genetic variability of field‐sampled founding populations to maximize the number of *csd* alleles and therefore maximize population increase during augmentation and after introduction. Also, it has been suggested that species with two‐locus CSD in the wild can degrade to single‐locus CSD in a biological control population after bottlenecks during importation, lowering the potential impact on the pest (De Boer et al., 2012). Moreover, some rearing practices could be detrimental for parasitoids with CSD, such as fragmenting laboratory populations or generating isofemale lines with the intention of mixing them only before release (Hoddle, Warner, Steggall, & Jetter, 2015; Roush & Hopper, 1995). These practices could result in subpopulations with few CSD alleles where production of diploid males will increase, leading to the extinction of some lines, and eventually, the loss of genetic variability in the population as a whole. On the other hand, it points to the fact that maintaining greater genetic variability could result in a more rapid population growth during captive rearing and a better chance of successful establishment in the field once released (Mackauer, 1976; Stouthamer, Luck, & Werren, 1992).

Therefore, we suggest that the practice of biological control can greatly benefit from an understanding of the genetic processes in small populations, including sex determination mechanisms, particularly in hymenopteran parasitoids, and that such studies should be implemented in the early stages of a program to achieve maximum success and efficacy.

## DATA ACCESSIBILITY

Data are available from the Dryad Digital Repository: https://doi.org/10.5061/dryad.1vm8k
