# Supplementary material for: Effects of inbreeding on a gregarious parasitoid wasp with complementary sex determination
Source: Evol Appl. 2017 Oct 13;11(2):243–53. doi: 10.1111/eva.12537 (PMC5775491; doi:10.1111/eva.12537)
Supplement: Supplementary file 1 [file EVA-11-243-s001.docx]

**Supplementary Information**

**Table S1.** Details of the number of females genotypes from the different lines according to crossing treatment (inbred – outbred) and generation (G1 = generation 1; G3 = generation 3; G5 = generation 5).

| Crossing treatment | Generation | Line code | Number of females genotyped | Total per generation |
| --- | --- | --- | --- | --- |
| Inbred | G1 | ENDO 01 | 1 | 42 |
|  |  | ENDO 02 | 1 |  |
|  |  | ENDO 03 | 1 |  |
|  |  | ENDO 06 | 1 |  |
|  |  | ENDO 09 | 1 |  |
|  |  | ENDO 10 | 1 |  |
|  |  | ENDO 11 | 1 |  |
|  |  | ENDO 14 | 1 |  |
|  |  | ENDO 15 | 1 |  |
|  |  | ENDO 16 | 1 |  |
|  |  | ENDO 17 | 1 |  |
|  |  | ENDO 18 | 1 |  |
|  |  | ENDO 19 | 1 |  |
|  |  | ENDO 20 | 1 |  |
|  |  | ENDO 21 | 1 |  |
|  |  | ENDO 22 | 1 |  |
|  |  | ENDO 23 | 1 |  |
|  |  | ENDO 24 | 1 |  |
|  |  | ENDO 25 | 1 |  |
|  |  | ENDO 26 | 1 |  |
|  |  | ENDO 27 | 1 |  |
|  |  | ENDO 30 | 1 |  |
|  |  | ENDO 31 | 1 |  |
|  |  | ENDO 32 | 1 |  |
|  |  | ENDO 33 | 1 |  |
|  |  | ENDO 34 | 1 |  |
|  |  | ENDO 35 | 1 |  |
|  |  | ENDO 37 | 1 |  |
|  |  | ENDO 39 | 1 |  |
|  |  | ENDO 41 | 2 |  |
|  |  | ENDO 42 | 1 |  |
|  |  | ENDO 43 | 1 |  |
|  |  | ENDO 44 | 1 |  |
|  |  | ENDO 47 | 1 |  |
|  |  | ENDO 48 | 1 |  |
|  |  | ENDO 49 | 2 |  |
|  |  | ENDO 50 | 1 |  |
|  |  | ENDO 51 | 1 |  |
|  |  | ENDO 52 | 1 |  |
|  |  | ENDO 53 | 1 |  |
|  | G3 | ENDO 03 | 2 | 40 |
|  |  | ENDO 13 | 3 |  |
|  |  | ENDO 14 | 2 |  |
|  |  | ENDO 17 | 4 |  |
|  |  | ENDO 18 | 2 |  |
|  |  | ENDO 23 | 2 |  |
|  |  | ENDO 24 | 2 |  |
|  |  | ENDO 32 | 3 |  |
|  |  | ENDO 37 | 2 |  |
|  |  | ENDO 40 | 4 |  |
|  |  | ENDO 42 | 1 |  |
|  |  | ENDO 45 | 3 |  |
|  |  | ENDO 46 | 2 |  |
|  |  | ENDO 48 | 4 |  |
|  |  | ENDO 50 | 3 |  |
|  |  | ENDO 51 | 1 |  |
|  | G5 | ENDO 13 | 2 | 30 |
|  |  | ENDO 15 | 2 |  |
|  |  | ENDO 17 | 7 |  |
|  |  | ENDO 18 | 6 |  |
|  |  | ENDO 23 | 3 |  |
|  |  | ENDO 37 | 3 |  |
|  |  | ENDO 45 | 2 |  |
|  |  | ENDO 48 | 2 |  |
|  |  | ENDO 50 | 3 |  |
| Outbred | G1 | EXO 01 | 2 | 40 |
|  |  | EXO 02 | 1 |  |
|  |  | EXO 03 | 1 |  |
|  |  | EXO 05 | 1 |  |
|  |  | EXO 08 | 1 |  |
|  |  | EXO 09 | 1 |  |
|  |  | EXO 12 | 1 |  |
|  |  | EXO 13 | 1 |  |
|  |  | EXO 14 | 1 |  |
|  |  | EXO 15 | 1 |  |
|  |  | EXO 17 | 1 |  |
|  |  | EXO 18 | 1 |  |
|  |  | EXO 19 | 1 |  |
|  |  | EXO 21 | 1 |  |
|  |  | EXO 23 | 1 |  |
|  |  | EXO 25 | 1 |  |
|  |  | EXO 26 | 1 |  |
|  |  | EXO 29 | 1 |  |
|  |  | EXO 30 | 1 |  |
|  |  | EXO 31 | 1 |  |
|  |  | EXO 32 | 1 |  |
|  |  | EXO 37 | 2 |  |
|  |  | EXO 39 | 1 |  |
|  |  | EXO 40 | 1 |  |
|  |  | EXO 41 | 1 |  |
|  |  | EXO 43 | 1 |  |
|  |  | EXO 45 | 1 |  |
|  |  | EXO 47 | 1 |  |
|  |  | EXO 48 | 2 |  |
|  |  | EXO 49 | 1 |  |
|  |  | EXO 50 | 1 |  |
|  |  | EXO 51 | 1 |  |
|  |  | EXO 53 | 1 |  |
|  |  | EXO 54 | 1 |  |
|  |  | EXO 56 | 1 |  |
|  |  | EXO 57 | 1 |  |
|  |  | EXO 58 | 1 |  |
|  | G3 | EXO 01 | 1 | 40 |
|  |  | EXO 02 | 1 |  |
|  |  | EXO 04 | 1 |  |
|  |  | EXO 05 | 1 |  |
|  |  | EXO 07 | 2 |  |
|  |  | EXO 08 | 1 |  |
|  |  | EXO 13 | 1 |  |
|  |  | EXO 15 | 1 |  |
|  |  | EXO 17 | 1 |  |
|  |  | EXO 19 | 2 |  |
|  |  | EXO 21 | 1 |  |
|  |  | EXO 24 | 1 |  |
|  |  | EXO 26 | 1 |  |
|  |  | EXO 28 | 1 |  |
|  |  | EXO 29 | 1 |  |
|  |  | EXO 30 | 2 |  |
|  |  | EXO 31 | 1 |  |
|  |  | EXO 33 | 1 |  |
|  |  | EXO 36 | 1 |  |
|  |  | EXO 37 | 2 |  |
|  |  | EXO 38 | 1 |  |
|  |  | EXO 42 | 1 |  |
|  |  | EXO 44 | 1 |  |
|  |  | EXO 47 | 3 |  |
|  |  | EXO 48 | 2 |  |
|  |  | EXO 50 | 1 |  |
|  |  | EXO 51 | 1 |  |
|  |  | EXO 54 | 1 |  |
|  |  | EXO 56 | 1 |  |
|  |  | EXO 57 | 2 |  |
|  |  | EXO 58 | 2 |  |
|  | G5 | EXO 01 | 1 | 40 |
|  |  | EXO 04 | 1 |  |
|  |  | EXO 05 | 1 |  |
|  |  | EXO 07 | 2 |  |
|  |  | EXO 08 | 2 |  |
|  |  | EXO 12 | 1 |  |
|  |  | EXO 13 | 2 |  |
|  |  | EXO 15 | 2 |  |
|  |  | EXO 17 | 1 |  |
|  |  | EXO 19 | 2 |  |
|  |  | EXO 21 | 2 |  |
|  |  | EXO 26 | 1 |  |
|  |  | EXO 28 | 2 |  |
|  |  | EXO 29 | 2 |  |
|  |  | EXO 30 | 1 |  |
|  |  | EXO 31 | 2 |  |
|  |  | EXO 33 | 1 |  |
|  |  | EXO 37 | 1 |  |
|  |  | EXO 42 | 1 |  |
|  |  | EXO 44 | 2 |  |
|  |  | EXO 48 | 2 |  |
|  |  | EXO 49 | 2 |  |
|  |  | EXO 51 | 1 |  |
|  |  | EXO 54 | 2 |  |
|  |  | EXO 57 | 2 |  |
|  |  | EXO 58 | 1 |  |

**Table S2.** Description of the additional six microsatellite markers tested with fluorescent markers for *Mastrus ridens* and subsequent genotyping: primer sequences, size range, repeat motif and number of alleles (Na) were based on the genotyping of 232 individuals from both lines.

| Name | Primer sequence (5’ – 3’) | Size range (pb) | Fluorescent dye | Repeat motif | Na |
| --- | --- | --- | --- | --- | --- |
| MR028 | F: CAACGAATAGAGATACCCCACG  R: CACTCGCTTATCGCTCTCAG | 142-160 | FAM | (AC)18 | 4 |
| MR052 | F: CTGCTGCGTTTGGTTTACG  R: AGAACACGAGTAATCGAGAGTTCG | 230-232 | VIC | (AG)17 | 2 |
| MR060 | F: ATTATACGACCGGCTGATGG  R: ATTGATGTCTGCTCTTCGCC | 243-247 | NED | (AC)13 | 3 |
| MR089 | F: AGCGAGAGAGCGAATGAAAC  R: CCACCTCTCTTACTCGACCG | 337-339 | NED | (AG)18 | 2 |
| MR094 | F: CGTGCACCTCTTCGTACTCC  R: CCAAAAATTGCAAAGCAACC | 378-394 | FAM | (ACG)13 | 2 |
| MR106 | F: CCCTCTGCTTTGAAGCTTTG  R: CGAGAGAGGGAGAACGGAG | 396-402 | VIC | (AC)13 | 2 |

**Table S3**. Means and standard errors of life history traits for *M. ridens* and experimental numbers according to crossing treatment and generation.

|  |  | inbred | | |  | outbred | | |
| --- | --- | --- | --- | --- | --- | --- | --- | --- |
| Trait | Generation | N | Mean | SE |  | N | Mean | SE |
| Parasitism | 1 | 48 | 0.24 | 0.19 |  | 47 | 0.20 | 0.22 |
|  | 2 | 29 | 0.32 | 0.17 |  | 30 | 0.30 | 0.18 |
|  | 3 | 11 | 0.44 | 0.24 |  | 31 | 0.34 | 0.20 |
|  | 4 | 9 | 0.30 | 0.17 |  | 27 | 0.41 | 0.23 |
|  | 5 | 8 | 0.26 | 0.17 |  | 25 | 0.35 | 0.23 |
| Fecundity: total adult offspring | 1 | 48 | 12.46 | 1.6 |  | 47 | 10.36 | 1.7 |
|  | 2 | 30 | 18.93 | 2.2 |  | 30 | 17.20 | 2.3 |
|  | 3 | 11 | 18.45 | 2.1 |  | 31 | 16.16 | 2.7 |
|  | 4 | 9 | 20.67 | 3.4 |  | 27 | 22.22 | 2.7 |
|  | 5 | 8 | 13.50 | 3.6 |  | 25 | 24.12 | 3.2 |
| Pupa – adult progeny survival | 1 | 35 | 0.83 | 0.02 |  | 30 | 0.82 | 0.03 |
|  | 2 | 23 | 0.72 | 0.04 |  | 27 | 0.79 | 0.04 |
|  | 3 | 11 | 0.88 | 0.04 |  | 27 | 0.81 | 0.03 |
|  | 4 | 8 | 0.78 | 0.05 |  | 25 | 0.77 | 0.04 |
|  | 5 | 7 | 0.85 | 0.03 |  | 22 | 0.78 | 0.04 |
| Longevity reproducing females | 1 | 40 | 22.1 | 1.5 |  | 34 | 20.8 | 1.5 |
|  | 2 | 27 | 22.6 | 1.2 |  | 28 | 20.4 | 1.5 |
|  | 3 | 11 | 18.2 | 2.4 |  | 27 | 18.2 | 1.9 |
|  | 4 | 8 | 26.4 | 2.0 |  | 25 | 22.0 | 2.2 |
|  | 5 | 7 | 22.9 | 2.6 |  | 22 | 24.8 | 2.1 |
| Longevity non reproducing females | 1 | 36 | 14.4 | 1.4 |  | 38 | 14.6 | 1.6 |
|  | 2 | 24 | 13.9 | 1.4 |  | 19 | 16.7 | 1.6 |
|  | 3 | 16 | 8.6 | 1.3 |  | 38 | 11.7 | 1.0 |
|  | 4 | 11 | 12.2 | 1.6 |  | 27 | 13.3 | 1.3 |
|  | 5 | 9 | 12.7 | 1.5 |  | 29 | 14.0 | 1.2 |
|  |  |  |  |  |  |  |  |  |
| Number of daughters | 1 | 48 | 3.5 | 0.7 |  | 47 | 3.4 | 0.7 |
|  | 2 | 30 | 3.8 | 1.2 |  | 30 | 7.7 | 1.5 |
|  | 3 | 11 | 4.3 | 1.6 |  | 31 | 6.2 | 1.3 |
|  | 4 | 9 | 4.9 | 1.5 |  | 27 | 7.2 | 1.4 |
|  | 5 | 8 | 5.1 | 2.2 |  | 25 | 7.8 | 1.7 |
| Number of sons | 1 | 48 | 9.0 | 1.2 |  | 47 | 6.9 | 1.3 |
|  | 2 | 30 | 15.2 | 1.7 |  | 30 | 9.3 | 1.5 |
|  | 3 | 11 | 14.2 | 2.0 |  | 31 | 10.0 | 2.0 |
|  | 4 | 9 | 15.8 | 3.1 |  | 27 | 15.0 | 2.2 |
|  | 5 | 8 | 8.4 | 2.0 |  | 25 | 16.3 | 2.8 |
|  |  |  |  |  |  |  |  |  |
| Sex ratio: proportion of males | 1 | 38 | 0.72 | 0.04 |  | 34 | 0.67 | 0.05 |
|  | 2 | 26 | 0.83 | 0.04 |  | 27 | 0.55 | 0.06 |
|  | 3 | 11 | 0.78 | 0.07 |  | 27 | 0.66 | 0.04 |
|  | 4 | 8 | 0.75 | 0.06 |  | 24 | 0.67 | 0.05 |
|  | 5 | 7 | 0.63 | 0.09 |  | 22 | 0.67 | 0.05 |
|  |  |  |  |  |  |  |  |  |
|  | Generation | Total males | Diploid | Proportion |  | Total males | Diploid | Proportion |
| Proportion Diploid males | 1 | 48 | 10 | 0.208 |  | 38 | 5 | 0.132 |
|  | 2 | 33 | 6 | 0.182 |  | 28 | 4 | 0.143 |
|  | 3 | 17 | 6 | 0.353 |  | 45 | 4 | 0.089 |
|  | 4 | 12 | 1 | 0.083 |  | 28 | 3 | 0.107 |

**Table S4**. GLM ANOVA results for female life history traits of *M. ridens* with two crossing protocols (inbred and outbred) for five generations.

| Trait | Source | Df | Deviance | Resid. df | Resid. dev. | F | Pr (>F) |
| --- | --- | --- | --- | --- | --- | --- | --- |
| a) Parasitism rate | Crossing treatment | 1 | 0.02 | 259 | 45.3 | 0.157 | 0.692 |
|  | Generation | 4 | 3.5 | 260 | 45.3 | 5.776 | < 0.001 |
|  | Interaction | 4 | 0.7 | 255 | 44.6 | 1.200 | 0.311 |
| b) Fecundity:  i) number of adult offspring | Crossing treatment | 1 | 0.06 | 221 | 1827.8 | 0.008 | 0.930 |
|  | Generation | 4 | 144.0 | 222 | 1827.8 | 4.504 | 0.002 |
|  | Interaction | 4 | 41.6 | 217 | 1786.1 | 1.302 | 0.270 |
| ii) Number of daughters | Crossing treatment | 1 | 44.4 | 221 | 1678.0 | 5.910 | 0.016 |
|  | Generation | 4 | 55.2 | 222 | 1722.3 | 1.839 | 0.123 |
|  | Interaction | 4 | 14.9 | 217 | 1663.0 | 0.497 | 0.738 |
| iii) Number of sons | Crossing treatment | 1 | 18.1 | 221 | 1653.4 | 2.426 | 0.121 |
|  | Generation | 4 | 92.3 | 222 | 1671.5 | 3.089 | 0.017 |
|  | Interaction | 4 | 71.3 | 217 | 1582.0 | 2.388 | 0.052 |
| c) Sex ratio | Treatment | 1 | 3.2 | 218 | 90.6 | 9.436 | 0.002 |
|  | Generation | 4 | 0.2 | 219 | 93.8 | 0.113 | 0.978 |
|  | Interaction | 4 | 2.7 | 214 | 87.9 | 2.027 | 0.092 |
| d) Pupa to adult survival | Crossing treatment | 1 | 0.01 | 209 | 41.5 | 0.037 | 0.848 |
|  | Generation | 4 | 1.2 | 210 | 41.5 | 1.602 | 0.175 |
|  | Interaction | 4 | 0.9 | 205 | 40.6 | 1.172 | 0.324 |
| e) Longevity  i) reproducing females | Crossing treatment | 1 | 5.5 | 220 | 911.1 | 1.411 | 0.236 |
|  | Generation | 4 | 34.2 | 221 | 916.6 | 2.191 | 0.071 |
|  | Interaction | 4 | 5.9 | 216 | 905.3 | 0.375 | 0.826 |
| ii) non reproducing females | Crossing treatment | 1 | 14.3 | 168 | 500.4 | 4.680 | 0.032 |
|  | Generation | 4 | 37.6 | 169 | 514.7 | 4.110 | 0.008 |
|  | Interaction | 4 | 3.0 | 165 | 497.5 | 0.324 | 0.808 |

Df: Degree freedom; Resid. Df: Residual degree freedom; Resid. dev: Residual deviance; Pr(>F): *P* values.

**Table S5.** GLM ANOVA results for life history traits of *Mastrus ridens* males according to ploidy (haploid and diploid), crossing treatment (inbred and outbred) and generation (1-5).

| Male Traits | Source | Df | Deviance | Resid. df | Resid. dev. | F | Pr(>F) |
| --- | --- | --- | --- | --- | --- | --- | --- |
| Number of daughters | Male ploidy | 1 | 37.1 | 219 | 1566.7 | 5.145 | 0.024 |
|  | Crossing treatment | 1 | 79.3 | 220 | 1603.7 | 10.999 | 0.001 |
|  | Generation | 4 | 84.2 | 221 | 1683.0 | 2.921 | 0.022 |
|  | Gen x cross | 4 | 25.7 | 215 | 1540.9 | 0.893 | 0.469 |
|  | Gen x Ploidy | 4 | 50.0 | 211 | 1490.9 | 1.736 | 0.143 |
|  | Cross x Ploidy | 1 | 0.1 | 210 | 1490.8 | 0.017 | 0.896 |
|  | Gen x Cross x Ploidy | 4 | 26.9 | 206 | 1463.8 | 0.935 | 0.445 |
|  |  |  |  |  |  |  |  |
| Number of sons | Male ploidy | 1 | 26.6 | 219 | 2000.8 | 3.142 | 0.078 |
|  | Crossing treatment | 1 | 0.4 | 220 | 2027.5 | 0.045 | 0.833 |
|  | Generation | 4 | 92.6 | 221 | 2027.8 | 2.733 | 0.030 |
|  | Gen x cross | 4 | 67.9 | 215 | 1933.0 | 2.003 | 0.095 |
|  | Gen x Ploidy | 4 | 29.6 | 211 | 1903.4 | 0.873 | 0.481 |
|  | Cross x Ploidy | 1 | 0.01 | 210 | 1903.4 | 0.002 | 0.969 |
|  | Gen x Cross x Ploidy | 4 | 52.6 | 206 | 1850.8 | 1.553 | 0.188 |
|  |  |  |  |  |  |  |  |
| Number of adult offspring | Male ploidy | 1 | 1.5 | 219 | 2381.4 | 0.162 | 0.688 |
|  | Crossing treatment | 1 | 19.2 | 220 | 2382.9 | 2.019 | 0.157 |
|  | Generation | 4 | 159.7 | 221 | 2402.2 | 4.195 | 0.003 |
|  | Gen x cross | 4 | 77.8 | 215 | 2303.6 | 2.043 | 0.090 |
|  | Gen x Ploidy | 4 | 48.2 | 211 | 2255.4 | 1.266 | 0.284 |
|  | Cross x Ploidy | 1 | 0.3 | 210 | 2255.1 | 0.031 | 0.861 |
|  | Gen x Cross x Ploidy | 4 | 50.6 | 206 | 2204.5 | 1.330 | 0.260 |
|  |  |  |  |  |  |  |  |
|  |  |  |  |  |  | Chi^2^ | Pr(>Chi) |
| Proportion of males with daughters | Male ploidy | 1 | 6.8 | 223 | 270.7 | 6.8 | 0.009 |
|  | Crossing treatment | 1 | 12.5 | 224 | 277.5 | 12.5 | < 0.001 |
|  | Cross x Ploidy | 1 | 0.8 | 222 | 269.9 | 0.8 | 0.363 |
| Proportion of males with granddaughters | Male ploidy | 1 | 0.8 | 118 | 104.1 | 0.8 | 0.386 |
|  | Crossing treatment | 1 | 7.4 | 117 | 96.7 | 7.4 | 0.007 |
|  | Cross x Ploidy | 1 | 3.2 | 116 | 93.5 | 3.2 | 0.072 |

Df: Degree freedom; Resid. Df: Residual degree freedom; Resid. dev: Residual deviance; Pr(>F) and Pr(>Chi): *P* values.
